# Proteomic Analysis Reveals Salt-Tolerant Mechanism in Soybean Applied with Plant-Derived Smoke Solution

**DOI:** 10.3390/ijms241813734

**Published:** 2023-09-06

**Authors:** Setsuko Komatsu, Taiki Kimura, Shafiq Ur Rehman, Hisateru Yamaguchi, Keisuke Hitachi, Kunihiro Tsuchida

**Affiliations:** 1 Faculty of Environment and Information Sciences, Fukui University of Technology, Fukui 910-8505, Japan; mtaiji71@gmail.com; 2 Department of Biology, University of Haripur, Haripur 22620, Pakistan; drshafiq@uoh.edu.pk; 3 Department of Medical Technology, Yokkaichi Nursing and Medical Care University, Yokkaichi 512-8045, Japan; h-yamaguchi@y-nm.ac.jp; 4 Institute for Comprehensive Medical Science, Fujita Health University, Toyoake 470-1192, Japan; hkeisuke@fujita-hu.ac.jp (K.H.); tsuchida@fujita-hu.ac.jp (K.T.)

**Keywords:** proteomics, salt stress, soybean, plant-derived smoke solution

## Abstract

Salt stress of soybean is a serious problem because it reduces plant growth and seed yield. To investigate the salt-tolerant mechanism of soybean, a plant-derived smoke (PDS) solution was used. Three-day-old soybeans were subjected to PDS solution under 100 mM NaCl for 2 days, resulting in PDS solution improving soybean root growth, even under salt stress. Under the same condition, proteins were analyzed using the proteomic technique. Differential abundance proteins were associated with transport/formaldehyde catabolic process/sucrose metabolism/glutathione metabolism/cell wall organization in the biological process and membrane/Golgi in the cellular component with or without PDS solution under salt stress. Immuno-blot analysis confirmed that osmotin, alcohol dehydrogenase, and sucrose synthase increased with salt stress and decreased with additional PDS solution; however, H^+^ATPase showed opposite effects. Cellulose synthase and xyloglucan endotransglucosylase/hydrolase increased with salt and decreased with additional PDS solution. Furthermore, glycoproteins decreased with salt stress and recovered with additional treatment. As mitochondrion-related events, the contents of ATP and gamma-aminobutyric acid increased with salt stress and recovered with additional treatment. These results suggest that PDS solution improves the soybean growth by alleviating salt stress. Additionally, the regulation of energy metabolism, protein glycosylation, and cell wall construction might be an important factor for the acquisition of salt tolerance in soybean.

## 1. Introduction

Soil salinization is a significant problem in the agricultural system, which is exhibited with climatic changes. Soil salinity is one of the factors contributing to reduced crop yields worldwide [1,2]. This is caused by the accumulation of water-soluble salts above threshold levels within the soil layer, which adversely affects seedling growth and seed yield [3]. The occurrence of soil salinization depends on various anthropogenic activities, farming practices, and soil types [4]. Saline soils are composed of soluble salts, such as sulfates/chlorides of sodium, magnesium, calcium, and potassium. Nitrate, carbonate, and bicarbonate ions are also present in saline soils. The pH and exchangeable sodium percentage of saline soils are less than 8 and 15, respectively [5]. All types of soil with diverse chemical, physical, and biological properties are affected by salinization [6]. With a growing global population and ever-increasing demand for food quality, the question of how to reduce soil salinity pressure, improve plant tolerance to salt stress, and ultimately increase crop yields needs to be addressed.

Soybean, which is an important source of protein and oil in the world, is highly sensitive to salt stress [7]. Soybean is classified as a salt-sensitive crop, and its yield can be reduced up to 40% by salt stress [8]. The shoot/root lengths and biomass are significantly reduced in cultivated soybean in comparison to wild types under salt stress [9]. Crops with different genotypes displayed different adaptation to salt stress, which is partly due to microorganisms in the rhizosphere [10]. Traditional breeding techniques combined with beneficial microorganisms were widely used to improve the salt tolerance of soybeans [11]. Seed priming and foliar application using jasmonic acid improved photosynthetic rate, transpiration rate, stomatal conductance, and chlorophyll fluorescence in salt-treated soybean [12]. These reports indicated that the development of salt-tolerant soybean is an important issue for improving soybean yields.

In contrast, fire generates smoke, ash, heat, and chemicals, all of which were recognized as germination signals for the growth of different species in fire-prone and fire-free habitats [13]. Plant-derived smoke (PDS) solution produced by burning plant parts such as the leaf, shoot, and stem facilitates the seed germination and plant growth process of over 1200 different plant species belonging to 80 different genera [14]. PDS solution is a material for promoting plant growth and development, which affects plant species from various habitats [15]. Butanolides, including karrikins and cyanohydrin, are the active compounds in PDS solution [16]. Karrikins are present in the smoke released by heating or burning plant material, and can stimulate the germination of dormant seeds. Additionally, karrikins have potential functions in mediating abiotic-stress tolerance in plants [17,18,19], and similar functions to strigolactones in the adaptation of plants to abiotic stress [20]. Because PDS solution has many more components, the effects against plant growth under abiotic stress are not completely clarified.

The combined effect of plant growth-promoting bacteria and PDS solution was more effective than the individual effect on rice [21]; however, they are both usually used individually to improve plant growth under saline conditions. In the case of soybean, PDS solution positively affected the post-germination growth [22,23]. Additionally, this solution enhanced soybean growth under flooding [24] and after flooding [25]. In contrast, soybean tolerance against salt stress with PDS solution has not been characterized. In this study, PDS solution is used to characterize salt-tolerant mechanisms in soybean. Based on the morphological results, proteomic analysis was performed using nano-liquid chromatography (LC) and mass spectrometry (MS)/MS to explore the tolerant mechanism for the positive effect on the growth of soybean treated with PDS solution under salt stress. Proteomic results were subsequently confirmed by immuno-blot and enzymatic analyses.

## 2. Results

### 2.1. Morphological Analysis of Soybean Treated with Plant-Derived Smoke Solution under Salt Stress

**Figure 1:**
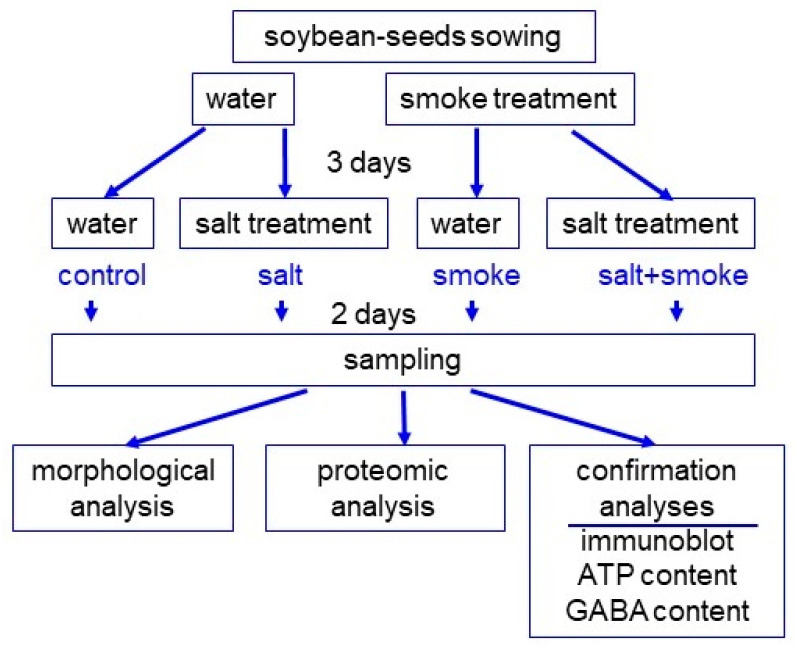
Experimental design to investigate the effects of PDS solution on soybean under salt stress. Seeds were sown and treated with or without 2000 ppm PDS solution. After 3 days of sowing, soybean seedlings were treated with 100 mM NaCl for 2 days. Soybean seedlings were analyzed with morphological and proteomic methods before being confirmed. For confirmatory experiments, immuno-blot and enzymatic analyses were used. All experiments were performed using 3 independent biological replicates. GABA means gamma-aminobutyric acid.

**Figure 2:**
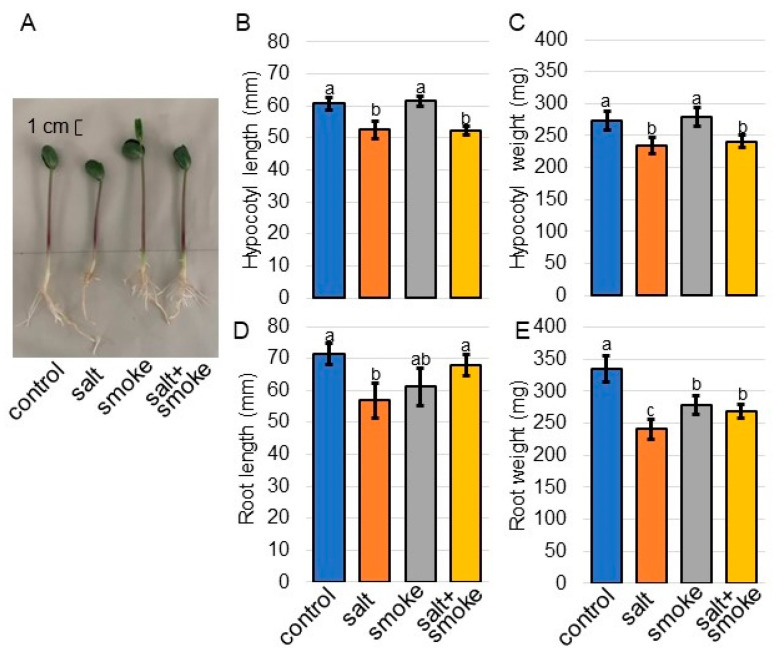
Morphological analysis of soybean treated with PDS solution under salt stress. Soybean seeds were sown and treated with or without 2000 ppm PDS solution. Three-day-old soybeans were treated for 2 days with or without salt stress. Four treatments were performed: control (blue), salt (orange), smoke (gray), and salt + smoke (yellow). Before morphological analysis, a photograph was taken (**A**). The bar in the picture indicates 1 cm. As morphological parameters, hypocotyl length (**B**), hypocotyl-fresh weight (**C**), main-root length (**D**), and total-root fresh weight (**E**) were analyzed at 5 days after sowing. The data are presented as mean ± SD from 5 independent biological replicates. Mean values in each point with different letters are significant according to one-way ANOVA followed by Tukey’s multiple comparisons (*p* < 0.05).

Morphological analysis was conducted to investigate the effects of plant-derived liquid smoke on soybean under salt stress. The concentration of the PDS solution was determined as 2000 ppm with previous reports, such as the dose-dependent experiments of maize [26] and chickpea [27], as well as a pre-experiment of soybean under salt stress. Soybean seeds were treated with 2000 ppm of the PDS solution, and 3-day-old seedlings were treated with 100 mM NaCl for 2 days (Figure 1). Morphological parameters such as hypocotyl length, hypocotyl fresh weight, main root length, and total root fresh weight were measured (Figure 2). All parameters decreased under salt stress; however, main root length and total root fresh weight increased with the application of the PDS solution, even if it was under salt stress (Figure 2). Additionally, root length (Figure 2D) and root weight (Figure 2E) showed similar trends. Based on these morphological results, soybean root was used for proteomic analysis.

### 2.2. Identification and Functional Investigation of Proteins in Soybean Treated with Plant-Derived Smoke Solution under Salt Stress

Gel-free/label-free proteomics was performed to investigate the cellular mechanisms in soybean growth by application of PDS solution under salt stress. Four types of treatments were performed: control, salt, smoke, and salt+smoke. After processing, the proteins extracted from soybean root were concentrated, reduced, alkylated, and digested (Table S1). After analysis by LC combined MS/MS, the relative abundance of proteins without (Table S2) or with (Table S3) PDS solution under salt stress was compared to the control. A total of 7318 proteins were identified by LC-MS/MS analysis. The proteomic results of all 12 samples from four different groups were compared by principal-component analysis, showing different accumulation patterns of proteins with four different types of treatments (Figure S1). This result indicated that salt stress significantly affected soybean proteins; however, this effect was recovered at the protein level by applying PDS solution, even under salt stress (Figure S1).

**Figure 3:**
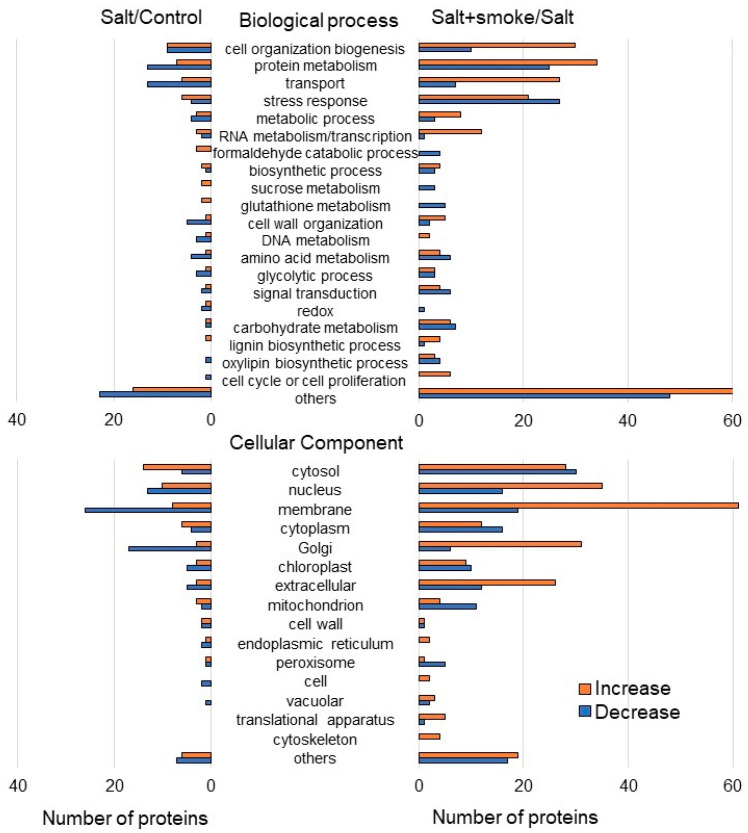
Functional categories of proteins with differential abundance in soybean root with PDS solution under salt stress. Four kinds of treatments (control, salt, smoke, salt + smoke) were performed. Proteins extracted from soybean root after treatment were enriched, reduced, alkylated, and digested. After analysis by LC combined with MS/MS, the relative abundance of proteins from without (Table S2) or with (Table S3) PDS solution under salt stress was compared to that of the control. Functional categories of changed proteins were determined using gene-ontology analysis. Red and blue columns indicate protein increases and decreases, respectively.

The abundance of 260 proteins differentially changed with the *p*-value < 0.05 and fold change > 1.5 and <0.66 in soybean roots under salt stress compared to the control condition (Table S2). Among the 260 proteins, 121 and 139 proteins increased and decreased, respectively, under salt stress compared to the control condition (Table S2 and Figure 3 left). In contrast, the abundance of another 659 proteins also differentially changed with the *p*-value < 0.05 and fold change > 1.5 and <0.66 in soybean roots applied with the PDS solution under salt stress compared to the salt condition (Table S3). Among these 659 proteins, 374 and 285 proteins increased and decreased, respectively, with the application of the PDS solution under salt stress compared to the salt condition (Table S3 and Figure 3 right). The functional category of identified proteins was obtained using gene ontology analysis (Figure 3). Differential abundance proteins between salt/control group and salt+smoke/salt group, which each indicate a difference in protein increase or decrease of two-fold or more, were associated with transport/formaldehyde catabolic process/sucrose metabolism/glutathione metabolism/cell wall organization in the biological process and membrane/Golgi in the cellular component. Furthermore, significantly changed proteins—which are the ten most abundant proteins—with differential abundance in soybean root with PDS solution under salt stress were listed (Table 1). Among them, alcohol dehydrogenase and glycosyltransferase significantly accumulated with salt stress and decreased with the addition of PDS solution even under salt stress (Table 1). To confirm the results obtained from the proteomic analysis, oppositely changed functional categories were further analyzed using immuno-blot and enzymatic analyses.

### 2.3. Immuno-Blot Analysis of Proteins Involved in Transport, Stress Response, Sucrose Metabolism, and Cell Wall Organization in Soybean with Application of Plant-Derived Smoke Solution under Salt Stress

**Figure 4:**
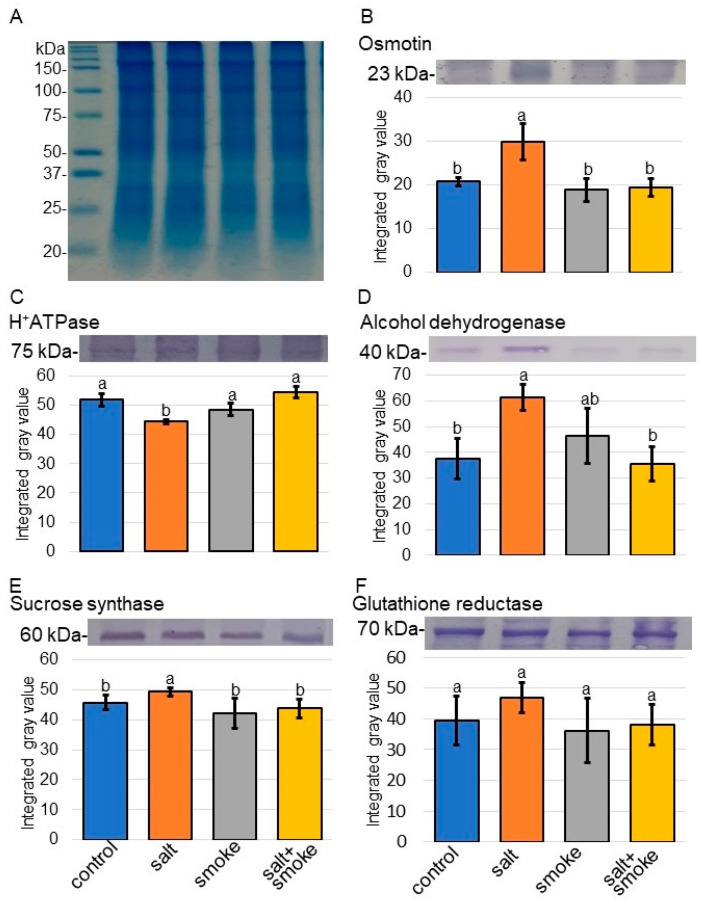
Immuno-blot analysis of the proteins involved in soybean treated with PDS solution under salt stress. Four treatments were performed: control (blue), salt (orange), smoke (gray), and salt+smoke (yellow). Proteins extracted from soybean root were separated on SDS-polyacrylamide gel by electrophoresis and stained with Coomassie-brilliant blue (**A**). A staining pattern with Coomassie-brilliant blue was used as a loading control. Proteins were transferred onto membranes. The membranes were cross-reacted with anti-osmotin (**B**), H^+^ATPase (**C**), alcohol dehydrogenase (**D**), sucrose synthase (**E**), and glutathione reductase (**F**) antibodies. The integrated densities of the bands were calculated using ImageJ software (version 1.8). The data are presented as mean ± SD from 3 independent biological replicates (Figures S2–S7). Statistical analysis is the same as in Figure 2.

**Figure 5:**
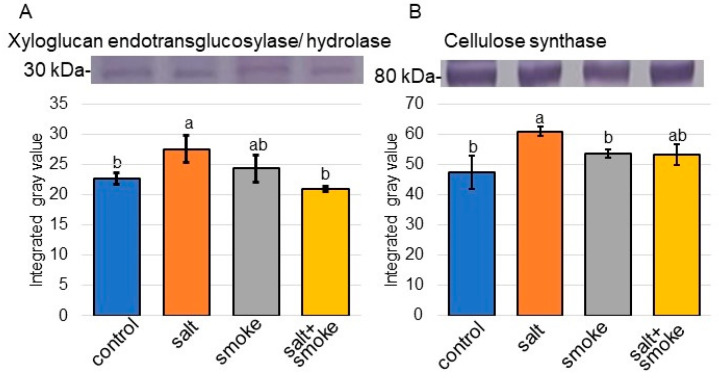
Immuno-blot analysis of the proteins involved in soybean treated with PDS solution under salt stress. Proteins blotted on the membrane were cross-reacted with anti-xyloglucan endotransglucosylase/hydrolase (**A**) and cellulose synthase (**B**) antibodies. A staining pattern with Coomassie-brilliant blue was used as a loading control (Figure S2). Three independent experiments were performed as biological replicates (Figures S8 and S9). Data analysis is the same as in Figure 4. Statistical analysis is the same as in Figure 2.

To better uncover the differential abundance proteins between the salt/control group and salt + smoke/salt group identified using proteomic analysis, immuno-blot analysis was performed. H^+^ATPase, alcohol dehydrogenase, sucrose synthase, glutathione reductase, xyloglucam endotransglucosylase/hydrolase, and cellulose synthase were selected as proteins associated with transport, formaldehyde-catabolic process, sucrose metabolism, glutathione metabolism, cell wall organization, and membrane, respectively (Figure 4 and Figure 5). Additionally, osmotin was used as a salt-stress response protein. Proteins extracted from soybean roots were separated on SDS-polyacrylamide gel by electrophoresis and transferred to polyvinylidene-difluoride membranes. The membranes were cross-reacted with anti-osmotin, H^+^ATPase, alcohol dehydrogenase, sucrose synthase, glutathione reductase, xyloglucam endotransglucosylase/hydrolase, and cellulose synthase antibodies. A staining pattern with Coomassie-brilliant blue was used as a loading control (Figure S2). The integrated densities of bands were calculated using ImageJ software (version 1.8) with triplicated immuno-blot results (Figures S3–S9). Immuno-blot analysis confirmed that osmotin, alcohol dehydrogenase, and sucrose synthase increased with salt stress and decreased with additional PDS solution; however, H^+^ATPase showed opposite effects (Figure 4). Cellulose synthase and xyloglucan endotransglucosylase/hydrolase increased with salt and decreased with additional PDS solution (Figure 5). The abundance of glutathione reductase did not change under salt stress as well as additional PDS solution. Furthermore, a comparison of alcohol-dehydrogenase abundances between proteomic and immuno-blot data showed similar trends in both (Figure S10). These results indicated that transport, formaldehyde-catabolic process, sucrose metabolism, and cell wall organization were regulated by PDS solution, even under salt conditions.

### 2.4. Lectin Blot Analysis of Proteins Involved in Soybean with the Application of Plant-Derived Smoke Solution under Salt Stress

**Figure 6:**
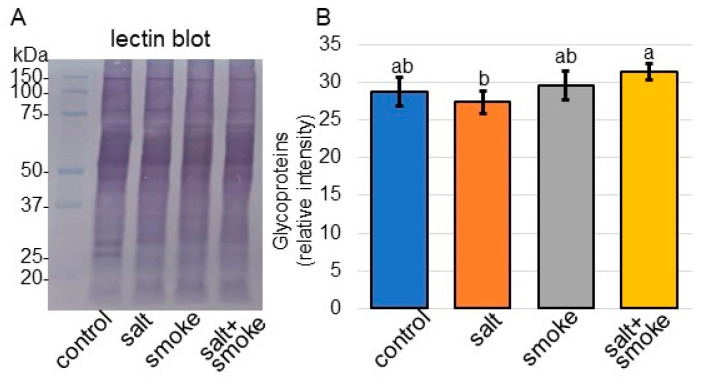
Lectin blot analysis of the proteins involved in soybean treated with PDS solution under salt stress. Proteins blotted on the membrane were cross-reacted with peroxidase-Concanavalin A (**A**). A staining pattern with Coomassie-brilliant blue was used as a loading control (Figure S2). The integrated densities of lectin blot were calculated using ImageJ software (version 1.8) (**B**). Data analysis is the same as in Figure 4. Statistical analysis is the same as in Figure 2.

Because proteins involved in the Golgi apparatus were oppositely changed in the cellular component with or without PDS solution under salt stress, the abundance of glycoproteins was analyzed (Figure 6). Proteins blotted on the membranes were cross-reacted with Concanavalin A. A staining pattern with Coomassie-brilliant blue was used as a loading control (Figure S2). The integrated densities of lectin blot (Figure 6A) were calculated using the triplicate immuno-blot results using ImageJ software (version 1.8) (Figure 6B). The number of glycoproteins was reduced by salt and restored by adding PDS solution (Figure 6).

### 2.5. The Contents of ATP and Gamma-Aminobutyric Acid in Soybean with Application of Plant-Derived Smoke Solution under Salt Stress

**Figure 7:**
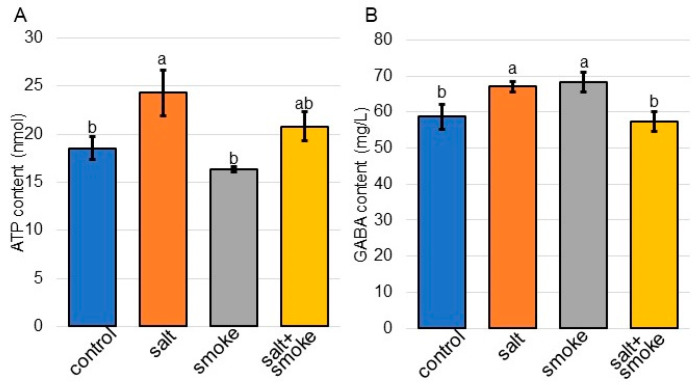
The contents of ATP and gamma-aminobutyric acid in soybean treated with PDS solution under salt stress. Soybean seeds were sown and treated with or without 2000 ppm PDS solution. Three-day-old soybeans were treated with or without salt stress for 2 days. Metabolites were extracted from the root. The ATP (**A**) and gamma-aminobutyric acid (**B**) contents were measured for each sample. Data analysis is the same as in Figure 4. Statistical analysis is the same as in Figure 2. GABA stands for gamma-aminobutyric acid.

Because proteins involved in mitochondrion were oppositely changed in the cellular component with or without PDS solution under salt stress, the contents of ATP and gamma-aminobutyric acid were analyzed (Figure 7). The contents of ATP and gamma-aminobutyric acid increased with salt and recovered with additional PDS solution (Figure 7).

## 3. Discussion

### 3.1. Cell Wall Organization Is Related to Salt-Tolerant Mechanism in Soybean with Plant-Derived Smoke Solution

Immuno-blot analysis confirmed that cellulose synthase and xyloglucan endotransglucosylase/hydrolase, which are membrane- and cell-wall-related proteins, increased with salt stress and decreased with additional PDS solution, even under salt stress (Figure 5). This result indicates that these two proteins increased by osmotic stress are recovered to the control level by additional PDS solution under salt stress. Using proteomic technique, xyloglucan endotransglucosylase/hydrolase significantly decreased with PDS solution in maize [26]. In the present study, the xyloglucan endotransglucosylase/hydrolase increased in soybean under salt stress decreased with the application of PDS solution, even under salt stress (Figure 5). In contrast, protein abundance and gene expression of the cell-wall-associated O-fucosyltransferase family proteins were higher in flooded soybean treated with PDS solution than in flooded soybean alone [25]. This indicated that PDS solution was an important factor contributing to the recovery of soybean from flooding stress. In the present study, cell-wall-related proteins in soybean indicated opposite results in osmotic stress, such as salt stress, compared to hypoxic stress, such as flooding stress.

Hemicelluloses are divided into 4 classes: xylans, mannans, beta-glucans with mixed linkages, and xyloglucans [28]. Xyloglucans are the most abundant hemicellulose in the primary cell walls of dicotyledonous plants [29]. Xyloglucan endoglycosidase/hydrolase modified cellulose and xyloglucan complex structures in the cell wall [30]. Xyloglucan endoglycosidase/hydrolase exhibits two catalytic functions: xyloglucan endohydrolase activity, which catalyzes the hydrolysis of xyloglucan, and xyloglucan endotransglucosylase activity, which cleaves and recombines xyloglucan chains [31]. *Xyloglucan endoglycosidase/hydrolase* genes have focused on abiotic stress responses such as osmotic, salt, and low-temperature stress responses. In *M. truncatula*, *MtXTH* genes responded to mercury, salt, and drought stress [32]. The expression of three homologous genes, *CaXTH1*, *CaXTH2*, and *CaXTH3,* were changed by drought, high salt, and low-temperature stress in pepper [33]. Overexpression of *CaXTH3* improved drought and salt tolerance in transgenic tomato [34], as well as the overexpression of *AtXTH31* enhanced flooding-stress tolerance in soybean [35]. These findings with the present results suggest that PDS solution confers tolerance to salt stress through the variation of xyloglucan endotransglucosylase/hydrolase in the cell wall of soybean.

In contrast, the cellulose, which is the main load-bearing component of plant cell walls, is synthesized in large quantities by cellulose synthase complexes. Cellulose synthase is a sophisticated molecular machinery that uses cortical microtubules as a steering device to navigate through cell membranes with its own catalytic activity [36]. Four components of the cellulose synthase machinery were identified: catalytic cellulose synthases [37], KORRIGAN [38], CSI1 [39], and CESA-COMPANION proteins, which are essential for the stability of CESAs and cortical microtubules against salt stress and fungal interaction [40]. Stress-sensing mechanisms enable rapid and controlled remodeling of the cell wall and mitigate perturbations on plant–cellulose synthesis by environmental cues to alleviate growth decline [41]. For example, the knockdown of a cellulose synthase gene *BoiCesA* affected leaf anatomy, cellulose content, and salt tolerance in broccoli [42]. These findings and the present results suggest that PDS solution confers tolerance to salt stress through the variation of cellulose modified by cellulose synthase of soybean.

### 3.2. Glycoprotein Folding Is Related to Salt-Tolerance Mechanism in Soybean Treated with Plant-Derived Smoke Solution

One of the most conserved translational modifications of protein is asparagine-linked glycosylation [43]. Following the tetradecyl glycan precursor, GlcNAc_2_Man_9_Glc_3_ (Glc for glucose, Man for mannose, and GlcNAc for N-acetylglucosamine) is transferred to specific asparagine residue on nascent polypeptide chains [44] by oligosaccharyltransferase complex [45], and the asparagine-linked oligomannosidic glycans are further trimmed and modified at the Golgi apparatus to synthesize mature N-glycans with associated protein secretion [46]. The first N-glycan processing event in the Golgi apparatus is catalyzed by two functionally redundant class I α-mannosidases, which cleave three α-1,2-mannosyl residues to generate substrate for CGL1/GnT1 in *Arabidopsis* [47]. GnT1/CGL1 catalyzes the GlcNAc addition necessary to remove two more Man residues and add another GlcNAc, xylose, and fucose residues to form a complex N-glycan structure [48]. In the present study, based on proteomic results, differential abundance proteins were found in the Golgi apparatus (Figure 2) and glucosyltransferase activity (Tables S2 and S3), which was confirmed by lectin blot analysis.

Moreover, N-glycosylation was associated with salt tolerance in *Arabidopsis* [46]. The adaptive response of plants to salt stress requires the maturation of N-glycan on associated proteins. Additionally, the failure of complex N-glycan biosynthesis causes salt sensitivity in *Arabidopsis* [49]. N-glycans play a crucial role in regulating stress-responsive protein levels, and several novel glycoproteins were involved in salt-stress tolerance in *Arabidopsis* [50]. In the present study, the abundance of glycoproteins decreased under salt stress and recovered with additional PDS solution, even under salt stress (Figure 6). These results, in conjunction with previous reports, suggest that glycoprotein folding in soybean is essential for recovery from salt stress. PDS solution might promote glycosylation and confer salt stress tolerance.

### 3.3. Energy Metabolism Iis Related to Salt-Tolerant Mechanism in Soybean Treated with Plant-Derived Smoke Solution

Sucrose synthase increased with salt stress and decreased with additional PDS solution; however, H^+^ATPase showed opposite effects (Figure 4). As mitochondrion-related events, the contents of ATP and gamma-aminobutyric acid increased with salt stress and recovered with additional treatment (Figure 7). Pre-treatments of PDS solution affected carbohydrate- and energy-related metabolic pathways such as starch/sucrose metabolism, galactose metabolism, glyoxylate metabolism, glycolysis, and tricarboxylic-acid cycle [51]. PDS solution promoted soybean-root growth through transcriptional enhancement with RNA polymerase II expression and energy production with ATPase accumulation [23]. These results indicates that PDS solution might relate to energy-production pathways.

Mitochondrial respiration is required during salt stress in plants because it provides ATP and reductants that fuel adaptive mechanisms such as compatible solute synthesis, ion exclusion, and reactive oxygen species detoxification [52]. Mitochondrial respiration is often located at the center of plant-metabolic networks because the tricarboxylic acid cycle links energy metabolism with both carbon and nitrogen metabolism [53]. The gamma-aminobutyric acid shunt activity is important for plant stress adaptation by regulating cytosolic pH, limiting reactive oxygen species production, regulating nitrogen metabolism, and bypassing steps in the tricarboxylic-acid cycle [54]. During salt exposure, key metabolic enzymes required for the cyclic operation of the tricarboxylic-acid cycle are physiochemically inhibited by salt. This inhibition is overcome by increased gamma-aminobutyric acid shunt activity, providing an alternative carbon source for mitochondria that bypasses salt-sensitive enzymes and promoting increased respiration in wheat leaves [55]. In this study, sucrose synthase, which breaks down sucrose for further use [56], increased under salt stress (Figure 4). Increased sucrose synthase, gamma-aminobutyric acid, and ATP under salt stress recovered at the control level with additional PDS solution (Figure 4 and Figure 6). The present results with previous findings suggest that PDS might contribute to the production of energy for plant growth, even under salt stress.

The content of gamma-aminobutyric acid and the activity of antioxidant enzymes increased, and the growth and development of soybeans were inhibited by salt stress [57]. In the present study using soybean, salt stress also increased gamma-aminobutyric acid (Figure 7B), which is consistent with previous findings. In contrast, gamma-aminobutyric acid in soybean increased with PDS solution alone (Figure 7B). Wheats responded to drought stress during the seedling stage, which related to reactive oxygen species scavenging systems and the activation of antioxidant enzymes, which were associated with activation of the gamma-aminobutyric acid shunt pathway and its production [58]. Additionally, the sonication or hydropriming treatments significantly improved the germination performance of wheat and enhanced gamma-aminobutyric acid metabolism to maintain the C:N metabolic balance under cold stress [59]. The present result combined with previous findings suggest that the contents of gamma-aminobutyric acid may increase when soybeans are stimulated by any compounds in PDS solution or perceive as them as stress.

## 4. Materials and Methods

### 4.1. Plant Material and Treatment

PDS solution was prepared using previous methods [24,60]. Briefly, the aerial parts of *Cymbopogon jwarncusa* L. were collected and washed with distilled water in order to remove the dust particles and were shade dried. A portion (333 g) of the semi-dried plant was smoldered in an airtight furnace. PDS was bubbled through 1 L of distilled water in a beaker to gain concentrated PDS solution, which was filtered through sterilized filter paper and diluted to 2000 ppm. Seeds of soybean (*Glycine max* L. cultivar Enrei) were sown on silica sand treated with or without 2000 ppm PDS solution. Three later of sowing, soybean seedlings were treated with or without 100 mM NaCl. Seedlings were maintained at 25 °C in a growth chamber illuminated with white-fluorescent light (200 µmol m^-2^ s^-1^, 16 h light period/day) and 70% relative humidity. For morphological analysis, root and hypocotyl from 5-day-old soybeans were used. Based on the morphological result, the root was used for proteomic and other biological analyses. More than three independent experiments were performed as biological replicates for all experiments. Independent biological replicates were sown on different days.

### 4.2. Protein Extraction

A portion (500 mg) of soybean roots was ground in 500 µL of lysis buffer consisting of 50 mM Tris-HCl, 150 mM NaCl, 1% Nonidet-P40, 0.1% SDS, and protease inhibitor (Nakalai Tesque, Kyoto, Japan) with a mortar and pestle. The suspension was centrifuged twice at 16,000× *g* for 20 min at 4 °C. Protein concentrations were determined using the method of Bradford [61], with bovine serum albumin as the standard.

### 4.3. Protein Enrichment, Reduction, Alkylation, and Digestion

Extracted proteins (100 µg) were adjusted to a final volume of 100 µL. Proteins were enriched, reduced, alkylated, and digested using previous methods [62] (Table S1).

### 4.4. Protein Identification Using nano-Liquid Chromatography Mass Spectrometry

The LC (EASY-nLC 1000; Thermo Fisher Scientific, San Jose, CA, USA) conditions as well as the MS (Orbitrap Fusion ETD MS; Thermo Fisher Scientific) conditions were described in the previous study [25] (Table S1).

### 4.5. Mass-Spectrometry Data Analysis

The MS/MS searches were carried out using MASCOT (version 2.6.2; Matrix Science, London, UK) and SEQUEST HT search algorithms against the UniprotKB *Glycine max* (SwissProt TreEMBL, TaxID = 3847, version 2021-05-14) using Proteome Discoverer 2.4 (version 2.4.0.305; Thermo Fisher Scientific). The workflow was described in the previous study [24] (Table S1).

### 4.6. Differential Analysis of Proteins using Mass-Spectrometry Data

Label-free quantification was performed with Proteome Discoverer 2.4 using precursor ions quantifiler nodes. Principal component analysis was also performed with Proteome Discoverer 2.4. For differential analysis of the relative abundance of peptides and proteins between samples, the free software PERSEUS (version 1.6.15.0) [63] was used. The workflow was described in the previous study [24] (Table S1).

### 4.7. Immuno-Blot Analysis

An SDS sample buffer consisting of 62.5 mM Tris-HCl (pH 6.8), 2% SDS, 5% dithiothreitol, 10% glycerol, and bromophenol blue was added to protein samples (Bio-Rad, Hercules, CA, USA). Quantified proteins (10 µg) were separated by electrophoresis on a 10% SDS-polyacrylamide gel and transferred onto a polyvinylidene difluoride membrane using a semidry transfer blotter. The blotted membrane was blocked for 5 min in Bullet Blocking One regent (Nacalai Tesque). After blocking, the membrane was cross-reacted with the primary antibodies for 30 min at room temperature. As primary antibodies, anti-osmotin [64], H^+^ATPase (Agrisera, Vannas, Sweden), alcohol dehydrogenase [65], sucrose synthase [66], glutathione reductase (Agrisera), xyloglucan endotransglucosylase/hydrolase (Agrisera), and cellulose synthase (Cosmo Bio, Tokyo, Japan) antibodies were used. As the secondary antibody, Anti-rabbit IgG conjugated with horseradish peroxidase (Bio-Rad) was used. For lectin blot, peroxidase-Concanavalin A (Seikagaku, Tokyo, Japan) was used. After 30 min incubation, signals were detected using tetramethylbenzidine solution (Nacalai Tesque) following protocol from the manufacturer. Coomassie-brilliant blue staining was used as a loading control. The integrated densities of bands were calculated using ImageJ software (version 1.8; National Institutes of Health, Bethesda, MD, USA).

### 4.8. Measurement of ATP Contents

The ATP content was measured using an ATP Colorimetric/Fluorometric Assay Kit (Biovision, Milpitas, CA, USA). A portion (150 mg) of samples was homogenized in 100 µL of the ADP assay buffer into a mortar and pestle. Extracts were centrifuged at 16,000× *g* for 10 min at 4 °C. For sample deproteinization and neutralization, the supernatant was treated with a Deproteinizing Sample Preparation Kit (Biovision). Extracts (50 µL) were added to 50 µL of a reaction mixture consisting of an ADP converter, ADP probe, ADP developer, and ADP assay buffer. After mixing and incubation for 30 min at 25 °C in the dark, the absorbance was measured at 570 nm.

### 4.9. Measurement of gamma-Aminobutyric Acid Contents

The gamma-aminobutyric acid enzymatic assay kit (Enzyme Sensor, Tsukuba, Japan) consisted of two solutions: solution I, containing 10 U/mL ascorbate oxidase, 0.8 U/mL glutamate oxidase, 1200 U/mL catalase, 10 U/mL peroxidase, and 0.8 mM 4-aminoantipyrine; and solution II, containing 2 U/mL gamma-aminobutyric acid, 0.8 mM N-ethyl-N-(2-hydroxy-3-sulfopropyl)-3-methylaniline sodium salt, 1 mM sodium 2-oxoglutarate, 2 mM pyridoxal phosphate, and 0.09% sodium azide. For the reaction, 0.5 mL of solution I was added to 50 µL of sample and incubated for 10 min at 30 °C. After incubation, 0.5 mL of solution II was added and incubated for 10 min at 30 °C. After additional incubation, the absorbance was measured at 555 nm. The contents of gamma-aminobutyric acid were determined with reference to the standard curve [67].

### 4.10. Statistical Analysis

The statistical significance of multiple groups was evaluated using a one-way ANOVA test. SPSS 20.0 (IBM, Chicago, IL, USA) statistical software was used for the evaluation of the results. A *p*-value of less than 0.05 was considered statistically significant.

## 5. Conclusions

PDS solution positively affected the post-germination growth of soybean [22,23], and this solution enhanced soybean growth during flooding [24] and after flooding [25]. In this study, PDS solution improved soybean growth, even under salt stress. Proteomic technique indicated that differential abundance proteins were associated with transport, stress response, sucrose metabolism, and cell wall organization with PDS solution under salt stress compared with salt stress only. Using biochemical techniques, the key findings were confirmed as follows: (i) osmotin, sucrose synthase, cellulose synthase, and xyloglucan endotransglucosylase/hydrolase increased with salt stress and decreased with additional PDS solution, while H^+^ATPase showed opposite effects; and (ii) glycoproteins decreased with salt stress and recovered with additional treatment, while the contents of ATP and gamma-aminobutyric acid showed opposite effects. These results suggest that PDS solution improves the soybean growth by stress reduction even under salt stress. Furthermore, the regulation of energy metabolism, protein glycosylation, and cell wall construction may be critical factors for the acquisition of salt tolerance in soybean. This research revealed that PDS solution acts on proteins to confer stress tolerance to soybeans against both flood and salt stress.

## Figures and Tables

**Table 1:** List of significantly changed proteins—which are the ten most abundant proteins—with differential abundance in soybean root with PDS solution under salt stress.

**Salt/Control**							
**Difference**	**Accession**	**Description**	**Cov**	**MP**	**AAs**	**MW**	**pI**
7.2714	K7MB33	Uncharacterized protein	13	7	838	92.4	4.69
4.0604	I1KF11	Dihydroorotase	24	6	346	38.4	6.98
4.0325	A0A0R0HMK4	Phosphatidylinositol-specific phospholipase C	8	2	421	46.1	7.50
3.6190	A0A0R0IZE4	40S ribosomal protein SA	48	15	310	33.9	5.26
3.1335	I1JY29	Alcohol dehydrogenase	27	6	381	41.2	6.00
3.0291	I1KC24	CBFD_NFYB_HMF	7	2	229	25.0	5.38
2.9526	K7MJ40	GOLD domain-containing protein	22	6	433	49.5	5.87
2.6089	I1L2Y7	Glycosyltransferase	8	3	473	52.7	6.02
2.6088	A0A0R0I6S7	Uncharacterized protein	11	3	461	52.9	7.66
2.4850	I1KN97	DUF3700 domain-containing protein	32	5	235	25.4	5.83
−3.3697	I1L0J1	CRAL-TRIO domain-containing protein	16	5	467	52.9	5.16
−3.4240	K7LMI3	K Homology domain-containing protein	11	6	794	84.2	5.06
−3.4587	I1M1F3	alpha-1,2-Mannosidase	5	2	610	67.8	7.09
−3.6234	A0A0R0GMV1	Cupin type-1 domain-containing protein	17	4	489	55.1	5.60
−3.7702	I1N036	Proliferating cell nuclear antigen	55	9	266	29.5	4.79
−3.8266	I1KWV5	Ubiquitin receptor RAD23	24	7	401	42.0	4.84
−3.8568	C6TBW8	Dihydrodipicolinate reductase	11	3	344	37.4	6.95
−3.9165	A0A0R0IL99	Glutamate--cysteine ligase	26	10	510	57.6	8.07
−3.9998	A0A0R0KVB4	Peptidase A1 domain-containing protein	29	7	427	46.4	8.32
−6.1145	A0A0R0KRW0	Cupin type-1 domain-containing protein	56	14	387	43.4	5.22
**Salt + Smoke/Salt**							
**Difference**	**Accession**	**Description**	**Cov**	**MP**	**AAs**	**MW**	**pI**
4.9048	C6TI83	BURP domain-containing protein	21	5	276	30.9	6.21
4.8781	I1MDT8	Serine decarboxylase	16	5	485	54.7	6.14
4.8530	I1JQB4	FAS1 domain-containing protein	15	3	453	50.1	6.46
4.7338	I1KWV5	Ubiquitin receptor RAD23	24	7	401	42.0	4.84
4.1979	A0A0R0EAL6	PKS_ER domain-containing protein	50	13	357	39.0	5.80
4.0866	K7KZF3	Fe2OG dioxygenase	12	3	314	35.9	6.09
3.8789	C6TBW8	Dihydrodipicolinate reductase	11	3	344	37.4	6.95
3.8521	I1K0I8	AT-hook motif nuclear-localized protein	14	2	327	33.4	8.95
3.8462	I1MDR1	Transmembrane protein 87B	6	2	516	58.2	6.46
3.8164	C6TJ36	Xyloglucan endotransglucosylase/hydrolase	7	2	302	34.2	5.76
−3.4548	C6T2R8	Glutathione S-transferase	31	8	216	25.0	5.57
−3.7336	A0A0R0KV96	Cytochrome P450	11	3	510	58.4	8.60
−3.8375	I1LYU9	Arginine biosynthesis bifunctional protein	14	3	464	48.5	6.37
−4.2521	Q42785	Nonsymbiotic hemoglobin	12	2	161	18.0	8.92
−4.3069	I1JE14	Glycosyltransferase	11	3	475	53.2	6.01
−4.3759	I1M4F8	Uncharacterized protein	12	2	302	32.9	5.29
−4.4426	A0A0R0F139	PKS_ER domain-containing protein	69	14	361	39.3	7.28
−5.4892	I1JY29	Alcohol dehydrogenase	27	6	381	41.2	6.00
−5.6909	Q9FQE8	Glutathione S-transferase	35	7	219	25.6	5.97
−6.1355	C6TMH1	ZnMc domain-containing protein	12	2	357	40.1	5.63

Four kinds of treatments (control, salt, smoke, salt + smoke) were performed. Proteins extracted from soybean root after treatment were enriched, reduced, alkylated, and digested. After analysis by LC combined with MS/MS, the relative abundance of proteins from without (Table S2) or with (Table S3) the PDS solution under salt stress was compared to that from the control.

## Data Availability

For MS data, RAW data, peak lists and result files have been deposited in the ProteomeXchange Consortium [68] via the jPOST [69] partner repository under data-set identifiers PXD032851.

## Supplementary Materials

The following supporting information can be downloaded at: https://www.mdpi.com/article/10.3390/ijms241813734/s1.
