# Supplementary material for: Proteomic Analysis Reveals Salt-Tolerant Mechanism in Soybean Applied with Plant-Derived Smoke Solution
Source: Int J Mol Sci. 2023 Sep 6;24(18):13734. doi: 10.3390/ijms241813734 (PMC10530690; doi:10.3390/ijms241813734)
Supplement: Supplementary file 1 [file ijms-24-13734-s001.zip › Figures S1-S10.pdf]

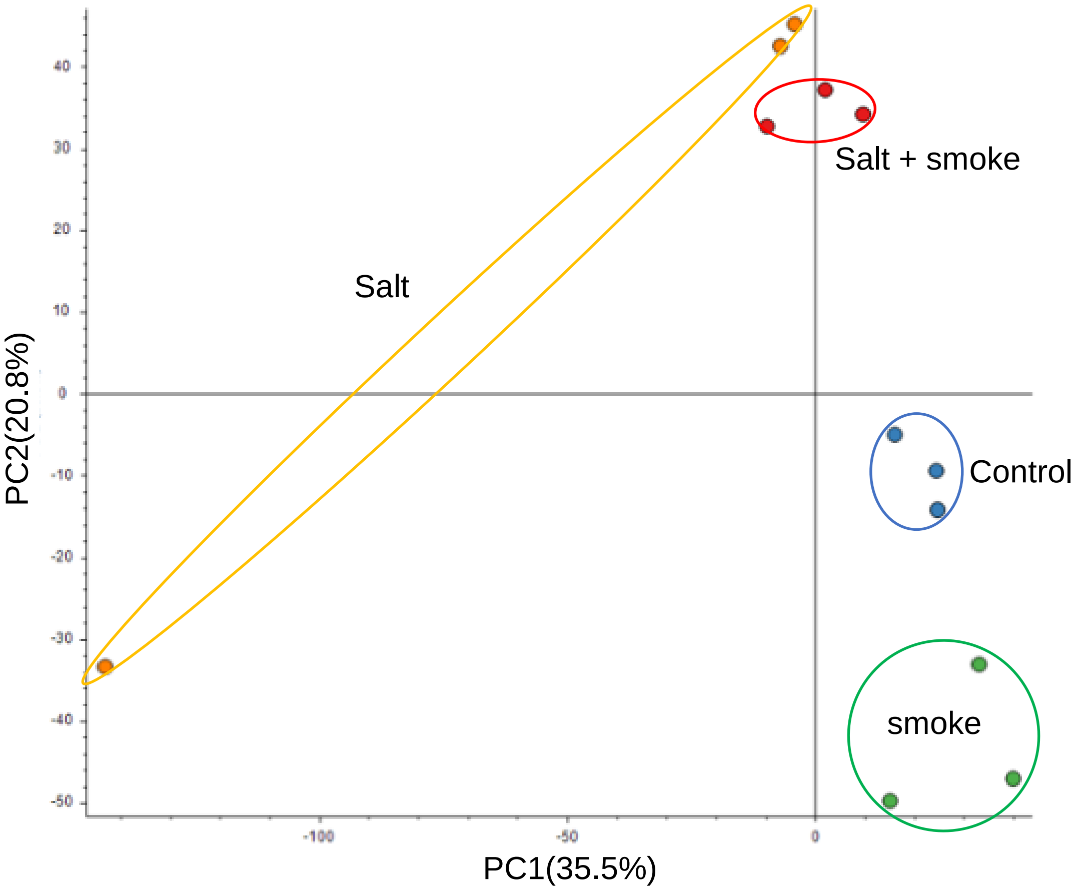

Figure S1. Overview of total proteomic data from 12 samples of soybean based on principal-component analysis. Three-day-old soybeans were exposed without or with plant-derived smoke solution under salt stress for 2 days. Proteomic analysis was performed with 3 independent biological replicates for each treatment. Principal-component analysis was performed with Proteome Discoverer 2.4.

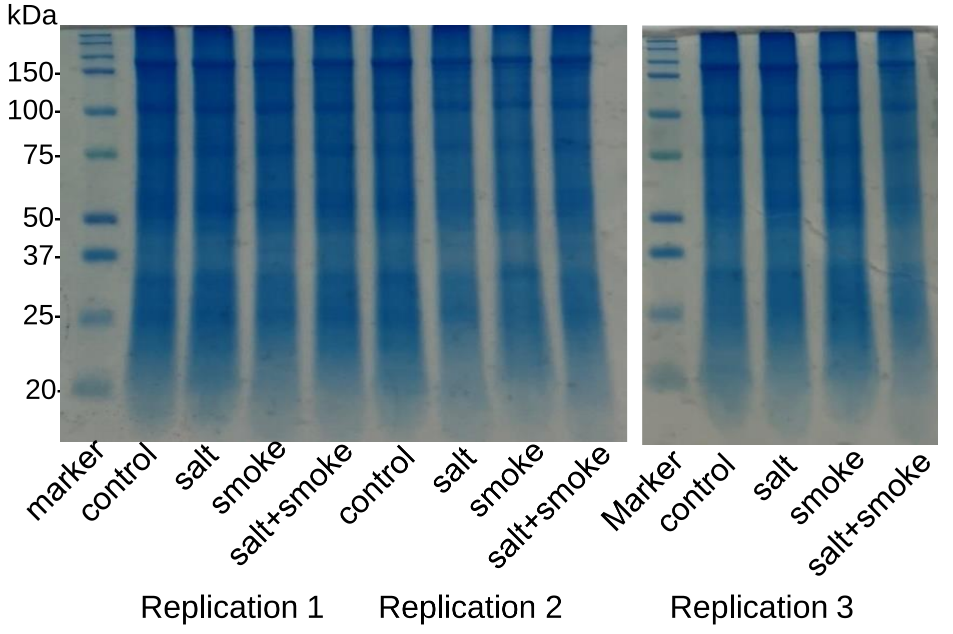

Figure S2. The Coomassie brilliant blue staining pattern of proteins used for immuno-blot analysis. Experiments were performed with biologically triplicates for each treatments. Quantified proteins (10 µg) from roots were separated by electrophoresis on a 10% SDS-polyacrylamide. Coomassie brilliant blue staining was used as a loading control.

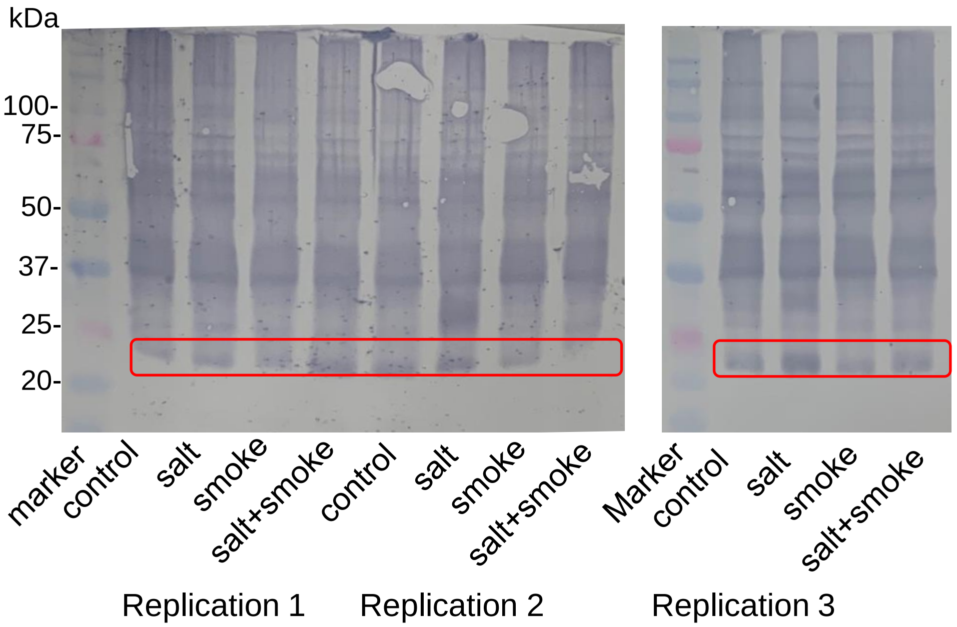

Figure S3 . Blots of the entire membrane with anti-osmotin antibody, which were used in Figure 4.

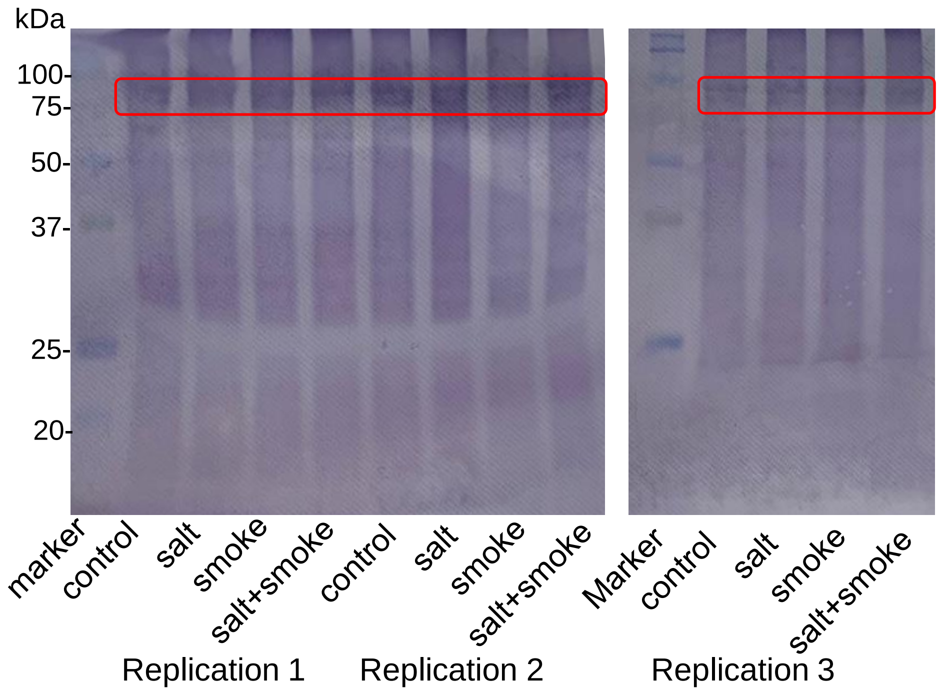

Figure S4 . Blots of the entire membrane with anti-H^+ATPase antibody, which were used in Figure 4.

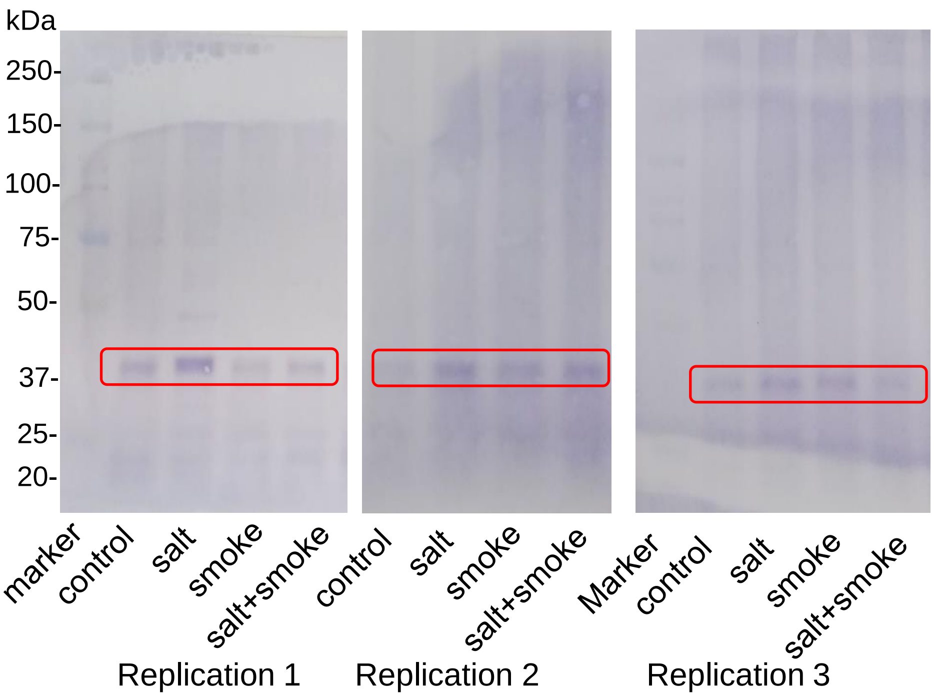

Figure S5 . Blots of the entire membrane with anti-alcohol dehydrogenase antibody, which were used in Figure 4.

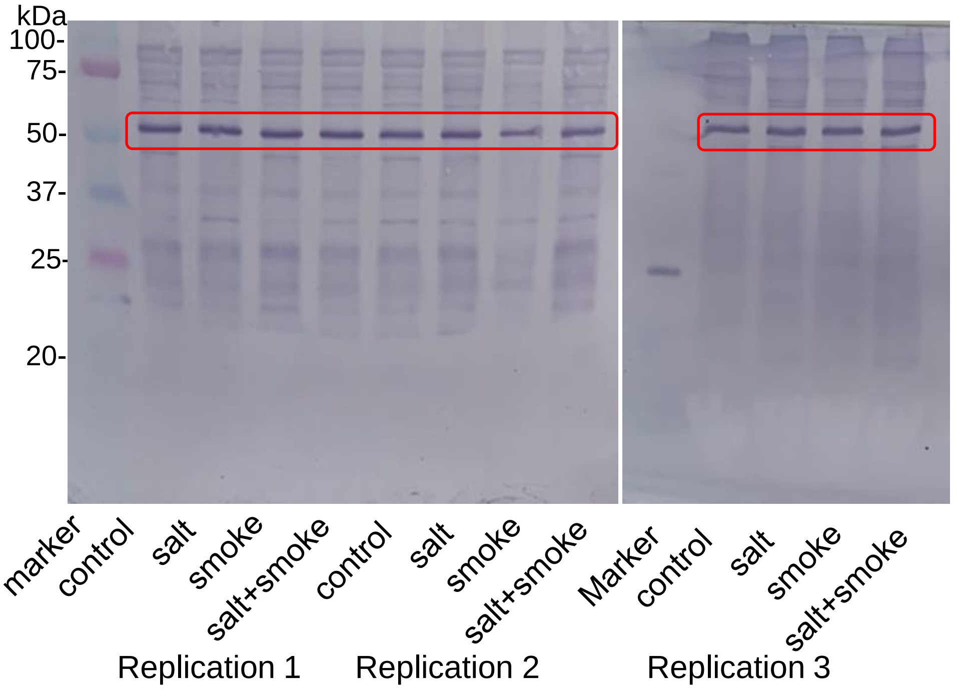

Figure S6. Blots of the entire membrane with anti-sucrose synthase antibody, which were used in Figure 4.

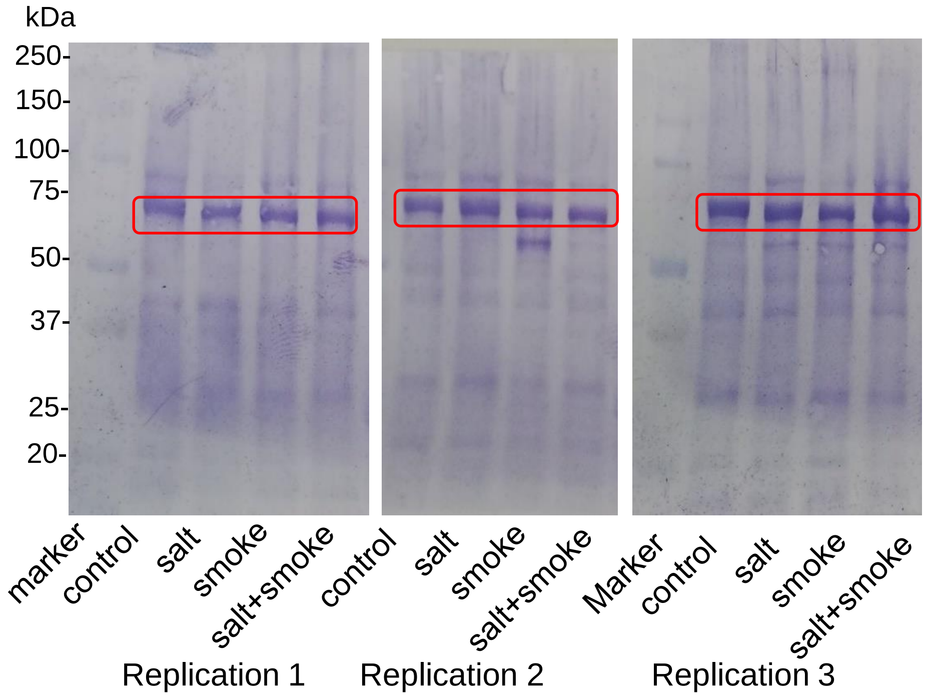

Figure S7. Blots of the entire membrane with anti-glutathione reductase antibody, which were used in Figure 4.

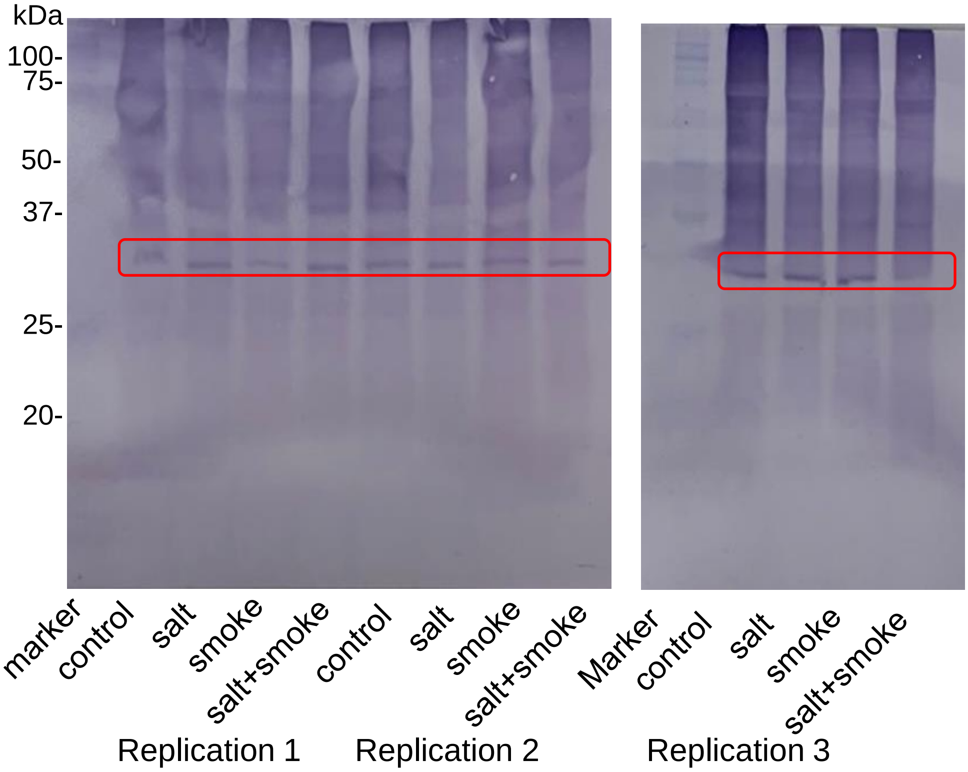

Figure S8. Blots of the entire membrane with anti-xyloglucan endotransglucosylase/ hydrolase antibody, which were used in Figure 5.

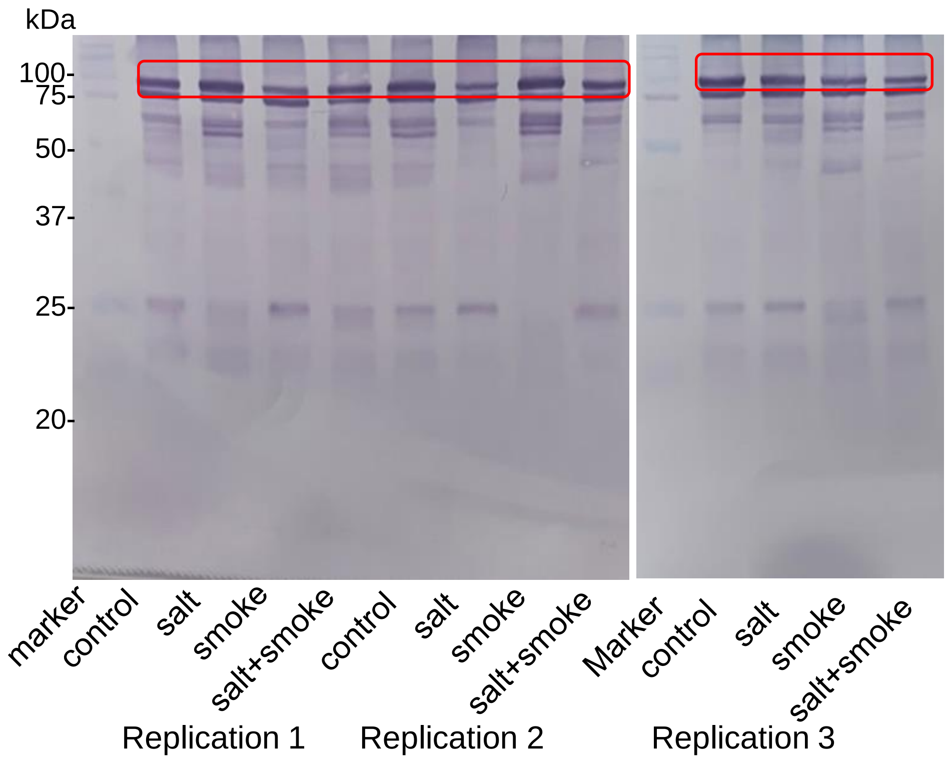

Figure S9. Blots of the entire membrane with anti-cellulose synthase antibody, which were used in Figure 5.

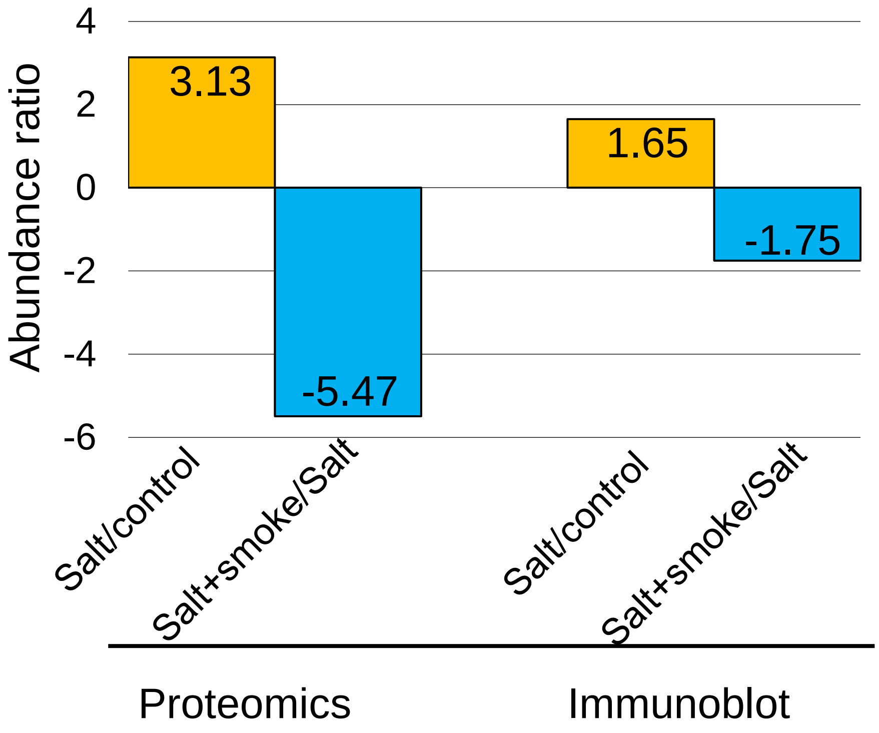

Figure S10. A bar plot between proteomic and immunoblot data of alcohol dehydrogenase.
