# Supplementary material for: Proteomic Analysis Reveals Salt-Tolerant Mechanism in Soybean Applied with Plant-Derived Smoke Solution
Source: Int J Mol Sci. 2023 Sep 6;24(18):13734. doi: 10.3390/ijms241813734 (PMC10530690; doi:10.3390/ijms241813734)
Supplement: Supplementary file 1 [file ijms-24-13734-s001.zip › Table S1.pdf]

Table S1. The experimental procedure of gel-free/label-free proteomics used in this research.

title	experimental procedure
Protein Enrichment, Reduction, Alkylation, and Digestion	Extracted proteins (100 μg) were adjusted to a final volume of 100 μL. Proteins were enriched, reduced, alkylated, and digested using previous methods (Komatsu et al., 2013). Methanol (400 μL) was added to each sample and mixed before addition of 100 μL of chloroform and 300 μL of water. After mixing and centrifugation at 20,000 x g for 10 min to achieve phase separation, the upper phase was discarded and 300 μL of methanol was added to the lower phase, and then centrifuged at 20,000 x g for 10 min. The pellet was collected as the soluble fraction. Proteins were resuspended in 50 mM ammonium bicarbonate, reduced with 50 mM dithiothreitol for 30 min at 56°C in the dark, and alkylated with 50 mM iodoacetamide for 30 min at 37°C in the dark. Alkylated proteins were digested with trypsin and lysyl endopeptidase (Wako, Osaka, Japan) at a 1:100 enzyme/protein ratio for 16 h at 37°C. Peptides were desalted with MonoSpin C18 Column (GL Sciences, Tokyo, Japan) and acidified with 1% trifluoroacetic acid.
Protein Identification using nano-Liquid Chromatography Mass Spectrometry	The liquid chromatography (LC) (EASY-nLC 1000; Thermo Fisher Scientific, San Jose, CA, USA) conditions as well as the mass spectrometry (MS) (Orbitrap Fusion ETD MS; Thermo Fisher Scientific) conditions were described in the previous study (Li et al., 2018). The peptides were loaded onto the LC system equipped with a trap column (Acclaim PepMap 100 C18 LC column, 3 μm, 75 μm ID x 20 mm; Thermo Fisher Scientific), equilibrated with 0.1% formic acid, and eluted with a linear acetonitrile gradient (0-35%) in 0.1% formic acid at a flow rate of 300 nL min^{-1}. The eluted peptides were loaded and separated on the column (EASY-Spray C18 LC column, 3 μm, 75 μm ID x 150 mm; Thermo Fisher Scientific) with a spray voltage of 2 kV (Ion Transfer Tube temperature: 275°C). The peptide ions were detected using MS in the data-dependent acquisition mode with the installed Xcalibur software (version 4.0; Thermo Fisher Scientific). Full-scan mass spectra were acquired in the MS over

	375-1,500 m/z with resolution of 120,000. The most intense precursor ions were selected for collision-induced fragmentation in the linear ion trap at normalized collision energy of 35%. Dynamic exclusion was employed within 60 sec to prevent repetitive selection of peptides.
Mass-Spectrometry Data Analysis	The MS/MS searches were carried out using MASCOT (version 2.6.2; Matrix Science, London, UK) and SEQUEST HT search algorithms against the UniprotKB *Glycine max* (SwissProt TreEMBL, TaxID=3847, version 2021-05-14) using Proteome Discoverer 2.4 (version 2.4.0.305; Thermo Fisher Scientific). The workflow was described in the previous study (Zhong et al., 2020). The workflow for both algorithms included spectrum files RC, spectrum selector, MASCOT, SEQUEST HT search nodes, percolator, ptmRS, and minor feature detector nodes. Oxidation of methionine was set as a variable modification and carbamidomethylation of cysteine was set as a fixed modification. Mass tolerances in MS and MS/MS were set at 10 ppm and 0.6 Da, respectively. Trypsin was specified as protease and a maximum of 2 missed cleavage was allowed. Target-decoy database searches used for calculation of false discovery rate, which was set at 1% for peptide identification.
Differential Analysis of Proteins using Mass Spectrometry Data	Label-free quantification was also performed with Proteome Discoverer 2.4 using precursor ions quantifiler nodes. Principal component analysis was also performed with Proteome Discoverer 2.4. For differential analysis of the relative abundance of peptides and proteins between samples, the free software PERSEUS (version 1.6.15.0) (Tyanova et al., 2016) was used. The workflow was described in the previous study (Zhong et al., 2020). Abundances of proteins and peptides abundances were transferred into log2 scale. Three biological replicates of each sample were grouped and a minimum of 3 valid values were required in at least one group. Normalization of the abundances was performed to subtract the median of each sample. Missing values were imputed based on a normal distribution (width = 0.3, down-shift = 1.8). Significance was assessed using Student's *t*-test analysis. Principle component analysis was performed with

	Proteome Discoverer 2.2. The sequences of the differentially accumulated proteins were subjected to a BLAST query against the gene ontology database (http://www.geneontology.org/).

References:

Komatsu, S.; Han, C.; Nanjo, Y.; Altaf-Un-Nahar, M.; Wang, K.; He, D.; Yang, P. Label-free quantitative proteomic analysis of abscisic acid effect in early-stage soybean under flooding. *J. Proteome Res.* **2013,** *12,* 4769-4784.

Li, X.; Rehman, S.U.; Yamaguchi, H.; Hitachi, K.; Tsuchida, K.; Yamaguchi, T.; Sunohara, Y.; Matsumoto, H.; Komatsu, S. Proteomic analysis of the effect of plant-derived smoke on soybean during recovery from flooding stress. *J. Proteomics* **2018,** *181,* 238–248.

Tyanova, S.; Temu, T.; Sinitcyn, P.; Carlson, A.; Hein, M.Y.; Geiger, T.; Mann, M.; Cox, J. The Perseus computational platform for comprehensive analysis of (prote) omics data. *Nat. Methods* **2016,** *13,* 731-740.

Zhong, Z.; Kobayashi, T.; Zhu, W.; Imai, H.; Zhao, R.; Ohno, T.; Rehman, S.U.; Uemura, M.; Tian, J.; Komatsu, S. Plant-derived smoke enhances plant growth through ornithine-synthesis pathway and ubiquitin-proteasome pathway in soybean. *J. Proteomics* **2020,** *221,* 103781.
